# Alexithymia and Suicide Risk in Psychiatric Disorders: A Mini-Review

**DOI:** 10.3389/fpsyt.2017.00148

**Published:** 2017-08-14

**Authors:** Domenico De Berardis, Michele Fornaro, Laura Orsolini, Alessandro Valchera, Alessandro Carano, Federica Vellante, Giampaolo Perna, Gianluca Serafini, Xenia Gonda, Maurizio Pompili, Giovanni Martinotti, Massimo Di Giannantonio

**Affiliations:** ^1^NHS, Department of Mental Health, Psychiatric Service of Diagnosis and Treatment, Hospital “G. Mazzini”, ASL 4, Teramo, Italy; ^2^Department of Neurosciences and Imaging, Chair of Psychiatry, Università degli Studi ‘G. d’Annunzio’ Chieti – Pescara, Chieti, Italy; ^3^New York Psychiatric Institute, Columbia University, New York City, NY, United States; ^4^Polyedra, Teramo, Italy; ^5^School of Life and Medical Sciences, University of Hertfordshire, Hatfield, United Kingdom; ^6^Villa S. Giuseppe Hospital, Hermanas Hospitalarias, Ascoli Piceno, Italy; ^7^Department of Mental Health, Psychiatric Service of Diagnosis and Treatment, Hospital “Madonna Del Soccorso”, NHS, San Benedetto del Tronto, Italy; ^8^Hermanas Hospitalarias, FoRiPsi, Department of Clinical Neurosciences, Villa San Benedetto Menni, Albese con Cassano, Italy; ^9^Department of Psychiatry and Neuropsychology, University of Maastricht, Maastricht, Netherlands; ^10^Department of Psychiatry and Behavioral Sciences, Leonard Miller School of Medicine, University of Miami, Miami, FL, United States; ^11^Department of Neuroscience, Rehabilitation, Ophthalmology, Genetics, Maternal and Child Health, Section of Psychiatry, University of Genova, Genova, Italy; ^12^Department of Psychiatry and Psychotherapy, Kutvolgyi Clinical Center, Semmelweis University, Budapest, Hungary; ^13^MTA-SE Neuropsychopharmacology Research Group, Hungarian Academy of Sciences and Semmelweis University, Budapest, Hungary; ^14^Department of Pharmacodynamics, Semmelweis University, Budapest, Hungary; ^15^Department of Neurosciences, Mental Health and Sensory Organs, Suicide Prevention Center, Sant’Andrea Hospital, Sapienza University of Rome, Rome, Italy

**Keywords:** alexithymia, suicide risk, psychiatric disorders, stress, prevention

## Abstract

It is well known that alexithymic individuals may show significantly higher levels of anxiety, depression, and psychological suffering than non-alexithymics. There is an increasing evidence that alexithymia may be considered a risk factor for suicide, even simply increasing the risk of development of depressive symptoms or *per se*. Therefore, the purpose of this narrative mini-review was to elucidate a possible relationship between alexithymia and suicide risk. The majority of reviewed studies pointed out a relationship between alexithymia and an increased suicide risk. In several studies, this relationship was mediated by depressive symptoms. In conclusion, the importance of alexithymia screening in everyday clinical practice and the evaluation of clinical correlates of alexithymic traits should be integral parts of all disease management programs and, especially, of suicide prevention plans and interventions. However, limitations of studies are discussed and must be considered.

## Introduction

It has been demonstrated in several studies that often alexithymic individuals may show significantly higher levels of psychological distress than non-alexithymics and may develop “functional” somatic symptoms and psychiatric symptoms such as anxiety and depression (1–3).

Moreover, it has been suggested that alexithymic subjects may scarcely respond to both pharmacotherapy and psychotherapy. The characteristic attributes of alexithymic behavior are predominantly manifest in social interactions with high emotional significance (4). The affect-avoiding interpersonal pattern behavior showed by such subjects is often maladaptive and may elicit disorders and conflicts in important relationships, lastly contributing the risk of development of psychiatric symptoms such as depression or anxiety, thus increasing the risk of suicide (5). Moreover, it has been demonstrated that alexithymia should be considered as a relative stable personality trait (6, 7), enhancing vulnerability to depressive symptoms (8, 9), and is generally associated with higher risk of death for several causes (accidents, injury, or violence) (10).

There is an increasing evidence that alexithymia may be considered a risk factor for suicide, even simply increasing the risk of development of depressive symptoms or *per se* (11). This evidence comes out from the results of several studies conducted on both general population and clinical samples of patients with psychiatric disorders or medical conditions.

Therefore, the aim of this narrative review was to elucidate the possible relationships between alexithymia and suicide risk, evaluating published studies on general population, suicide attempters, and subjects with psychiatric disorders [such as anxiety disorders, affective disorders, eating disorders (EDs), etc.]. Studies were identified searching the electronic databases such as MEDLINE, Embase, PsycINFO, and the Cochrane Library.

## Studies on Relationships Between Alexithymia and Suicide Risk in General Population

So far, two main and relevant studies on general population (both conducted in Finland) have been done to evaluate the relationships between alexithymia and suicide risk.

First, a sample of 1,722 participants (735 men and 987 women) were evaluated (12). In particular, the study focused on the hopelessness, a widely recognized risk factor for suicide (13). Researchers found that a lower financial status, poor personal health, and reduced working ability independently associated with hopelessness. Interestingly, the probability of moderate or severe hopelessness was considerably increased in individuals with alexithymia and suicidal ideation. They concluded that life discontent, depressive symptoms, alexithymia, and suicidal ideation were the most powerful factors associated with moderate to severe hopelessness, but causal association was not possible to argue as this was a cross-sectional study.

The second study analyzed a huge sample of 1,563 subjects in the Kuopio Depression Study (14). Interestingly, this study proved that the most common self-report measure of alexithymia [the 20-item Toronto Alexithymia Scale (TAS-20)] showed a large overlap with a largely used self-report inventory for measuring the severity of depression, the Beck Depression Inventory. The associations of alexithymia with levels of suicidal ideation across time “…were no longer independent when adjusted for concomitant changes in the level of depressive symptoms…” (14), as reported by author themselves. Thus, the conclusion was that depressive symptoms were the mediators between alexithymia and psychiatric morbidity, hence including suicide ideation.

All considered, studies on general population support the hypothesis of a relationship between alexithymia and suicidal ideation mediated by depressive symptoms.

## Studies on Relationships Between Alexithymia and Suicide Risk in Clinical Samples

Several studies on clinical samples of patients with psychiatric disorders have confirmed the hypothesis that alexithymia may increase the suicide risk, especially through the development of depressive symptoms.

### Studies on Suicide Attempters

The studies on possible risk factors in suicide attempters are very interesting due to several reasons. In fact, subjects who had a previous suicide attempt are at risk to reattempt suicide (15, 16). Therefore, the evaluation of alexithymia in such subject may be of particular interest to include it as a putative risk factor.

Interestingly, three studies have been conducted in suicide attempters. First (17), 50 suicide attempters were evaluated within 24 h after hospital admission, and most of the attempters showed depressive symptoms with near half regarded as positive for alexithymia. However, alexithymia was not more prevalent in suicide attempters than in non-suicidal subjects with depressive symptoms. Depressive symptoms and alexithymia were considerably associated, but the authors found no significant correlation between alexithymic traits and lethality of the suicide attempt or suicidal intent (17). The authors concluded that alexithymia in suicide attempters was associated with depressive symptoms, but not intrinsically with suicidal behavior. Moreover, in a study conducted on 100 suicide attempters compared to 60 healthy controls, the sample of suicide attempters did not show significantly higher scores on the alexithymia rating scale than healthy subjects, and alexithymia was not found to be a predominant trait of personality among suicide attempters or a significant predictive factor of suicidal behavior (18).

More recently, in a Greek study (19), researchers evaluated the possible relationship between alexithymia (measured using the Shalling-Sifneos Personality Scale Revised), depression, and serum lipids in 50 non-violent suicide attempters and found a significant association between alexithymia and depression in those who attempted suicide, but only alexithymia was correlated with higher serum triglyceride levels.

Overall, no direct link was found between alexithymia and suicidality among suicide attempters, while there was a confirmation that measures of alexithymia were strongly related to measures of depression.

### Studies on Patients with Psychiatric Disorders

The prevalence of alexithymia is quite high in subjects with psychiatric disorders (20). Therefore, the studies on relationships between alexithymia and suicide risk on clinical samples of patients with psychiatric disorders are very interesting as alexithymia may predispose to their development or worsen an existing one (8, 9, 21, 22).

#### Studies on Patients with Anxiety Disorders (ADs)

The presence of alexithymic traits in patients with ADs may be a risk factor of suicide, simply worsening the AD itself *per se* or leading to the development of depressive symptoms or even a comorbid clinically relevant major depressive episode (MDE) (23–25).

Concerning obsessive–compulsive disorder (OCD), it has been found that alexithymia and depressive symptoms were significantly correlated in subjects with OCD (11). In a study on 86 patients with OCD, alexithymia was associated with higher suicide risk, especially in patient with poor or absent insight and with a greater disorder severity (26). In this study, the “Difficulty in Identifying Feelings” (DIF) dimension of TAS-20 was positively correlated with higher scores on the Scale for Suicide Ideation (SSI). The relationships between alexithymia and higher suicide risk were also confirmed in another study that pointed out that alexithymic patients showed higher suicidal ideation than non-alexithymic patients, and this was associated with lower high-density lipoprotein (HDL) cholesterol levels and with the DIF subscale of TAS-20, regardless of depressive symptoms (27). More recently, De Berardis et al. (28) further demonstrated that alexithymic OCD subjects showed higher disorder severity, poor or absent insight, and exaggerated responsibility, all associated with suicide ideation, regardless of depressive symptoms. Again, in this research, the DIF subscale of TAS-20 was associated with higher SSI scores. Moreover, alexithymia and perfectionism have been found to contribute to higher suicidality in 81 patients with OCD (29). It should be noted that in most studies on patients with OCD, the links between alexithymia and depression were evaluated by comparing a self-rating scale (TAS-20) with an observer-rated scale, the Montgomery Åsberg Depression Rating Scale (MADRS). This must be accounted as a potential source of bias. In fact, when used as a modified self-report questionnaire, it was found that the MADRS focused on core symptoms of depression and was less influenced by maladaptive personality traits, and, therefore, this bias may be more marked with the observer-rated version of the scale (30).

Also in subjects with panic disorder (PD), a significant relationship between alexithymia and increased suicidal ideation has been found linked by a serum lipid dysregulation (31). In particular, the presence of lower HDL and higher very low-density lipoprotein cholesterol levels and DIF subscale of TAS-20 were associated with higher suicide ideation in a sample of 72 outpatients with PD. This positive correlation between alexithymia and increased suicidal ideation was substantially confirmed also in patients with generalized anxiety disorder (32).

Only one study directly evaluated alexithymia and suicide risk in posttraumatic stress disorder (PTSD), even if a link between alexithymia and posttraumatic symptoms has been showed in several studies (20, 33–37). Kusevic et al. (38) evaluated 127 veterans from the 1991 to 1995 war in Croatia, and results of the study suggested that alexithymia can be considered as a risk factor for attempted suicide among war veterans with PTSD. However, in this study, no independent measure of depression was used, and this may have biased results.

Overall, studies on ADs support the notion of a relationship between alexithymia and suicide ideation, even if in the absence of clinically relevant depressive symptoms.

#### Studies on Patients with Affective Disorders

Despite the great number of studies that have evaluated the presence and clinical correlates of alexithymia in affective disorders (AD) such as major depression (MD) (39–42), surprisingly, relatively few studies have directly investigated its relationships with suicide risk. Alexithymia may be a risk factor of suicide in adolescent depression especially in the presence of maladaptive early schematas (43). Concerning adults, De Berardis et al. (41) evaluated 145 drug-naive adult outpatients with a DSM-IV diagnosis of MD and found that alexithymic patients showed higher scores on SSI, thus indicating a higher suicide risk. In a linear regression model, lower HDL levels, DIF, and “Difficulty in Describing Feelings” (DDF) dimensions of TAS-20 were associated with higher suicide risk.

Moreover, Loas et al. (24) evaluated a mixed sample of patients with both mood (2 with bipolar depression and 35 with unipolar depression) and ADs and pointed out that alexithymia and, particularly, DIF dimension of TAS-20 had strong relationships with suicidal ideations and low cholesterol levels, low HDL levels, or low triglycerides levels in patients with both mood or ADs.

Recently, Serafini et al. (44) recruited 281 euthymic participants of whom 62.3% had MD and 37.7% had bipolar disorder and showed that such subjects may suffer from significant difficulties in processing sensory input (measure with the adolescent/adult sensory profile), which have been significantly linked with higher depression, impulsivity, alexithymia, and hopelessness. Lower registration of sensory input referring to hyposensitivity and sensation avoiding referring to hypersensitivity significantly correlated with higher alexithymia and, in particular, with DIF and DDF dimensions of TAS-20, accounting for higher impulsivity and hopelessness (that may be risk factors of suicide).

Taken together, all studies on patients with AD pointed out a relationship between alexithymia and suicide ideation, even if there are, to date, too few studies to draw definite conclusions.

#### Studies on Other Psychiatric Disorders

Positive correlations between alexithymia and increased suicide risk have been found in other several psychiatric disorders. Somatoform disorder patients with lifetime suicide attempts might have greater difficulties in identifying and describing emotions and are prone to intensely feel and express anger (45). Moreover, in patients with conversion disorder (CD), alexithymia is higher in suicide attempters (46).

Several evidences point out that alexithymia may be a risk factor of suicide in EDs. For example, Carano et al. (47) demonstrated that individuals with binge eating disorder (BED) and alexithymia showed higher suicide ideation than non-alexithymics, especially in the presence of subclinical depressive symptoms. Alpaslan et al. (48) evaluated 381 female students in Turkey and found that disordered eating attitudes (DEAs) were a quite common phenomenon in female students and the occurrence of alexithymia was related with a higher suicide risk in such subjects. However, no studies have been conducted to evaluate the association between alexithymia and increased suicide ideation in patients with anorexia nervosa and bulimia.

Moreover, several studies have pointed out that alexithymia may be a risk factor for suicide and self-harm in individuals with substance use disorders (SUDs) (49–51).

To date, just one study evaluated relationships between alexithymia and suicide risk in subjects with schizophrenia, and study results showed that the occurrence of alexithymic traits in schizophrenia was associated with more prominent suicide ideation and more serious depressive symptoms, independently by the severity of positive and negative symptoms (52).

In conclusion, it seems that alexithymia may be a putative risk factor for suicide in patients with CD, BED, DEAs, SUD, and schizophrenia, but further prospective studies are needed to further confirm this association.

## Other Limitations of Reviewed Studies

We have discussed some limitations of the reviewed studies above in the text. However, some other general limitations should be acknowledged.

The principal limitation of most studies on the relationships between alexithymia and suicide is that these are often cross-sectional and not prospective. Therefore, it may be difficult to interpret the identified associations and draw predictive conclusions.

Moreover, almost all reviewed studies employed the TAS-20, a self-report scale, to measure alexithymia. Even if the TAS-20 is a very useful tool in everyday “real-world” clinical practice to screen whether the subject is positive for alexithymia, it should be noted that findings based on self-report tools may be source of bias that in some cases may be poorly informative. Task-based studies should be preferred, but, to date, no task studies evaluating alexithymia were developed. However, future studies should employ the Toronto Structured Interview for Alexithymia (TSIA) (53), a reliable and valid structured interview for assessing the alexithymia construct, that can overcome the limitations of TAS-20 (54, 55).

Finally, the lack of control for adequate and comparable measures of depression and alexithymia or adequate statistical models of the interaction between depression and alexithymia (mixed models rather than ANCOVA) was a limitation of several reviewed studies, and this should be recognized.

## Conclusion

Taken together, the almost all studies have pointed out a significant relationship between alexithymia and an increased suicide risk.

These findings may be explained in several ways. One is in accordance to the theory proposed by Freyberger of acute “secondary alexithymia” as a response to stressful conditions (56). The “acute secondary alexithymia” (56) can be defined as a transient, state-dependent experience that may occur as a consequence of subjective distress and can decline when an acute episode of illness has resolved (57). As the presence of alexithymia may worsen an existing psychiatric or medical disease, this worsening may be related to the development of suicidal ideation *per se* or through the development of depressive symptoms or even a comorbid clinically relevant MDE (4). However, even if alexithymia may be a state-dependent phenomenon (i.e., alexithymia may worsen during an acute disease episode, as described above), it should be considered a relatively stable personality trait that may be also present even before the onset of a psychiatric disorder or a medical disease (6, 7). Thus, research findings may be also in accordance with the “stress-alexithymia hypothesis” (58): alexithymia may be a chronic condition [maybe with an onset during infancy or early adolescence and often in consequence of childhood abuse or neglect (49, 59)] characterized by a pronounced inflammatory state with an impaired hypothalamic–pituitary–adrenal axis reactivity to even mild to moderate life stressors (60). Therefore, it should be deemed as a chronic state reaction as a response to stressful conditions that may always complicate a psychiatric disorder or a medical disease (61).

In conclusion, the importance of alexithymia screening in everyday clinical practice and the evaluation of clinical correlates of alexithymic traits should be integral parts of all disease management programs and, especially, of suicide prevention plans and interventions. The TAS-20 is a very useful tool in everyday “real world” clinical practice to screen subject positive or not for alexithymia, even if it is a self-report scale with all limitations of such instruments. However, if a subject is positive for alexithymia on TAS-20, a careful evaluation should be conducted (also completing the assessment of alexithymia with the TSIA, if possible) concerning the presence of depressive symptoms and suicidal ideation.

## Author Contributions

We state that (1) all authors have read the paper and approved the data and the conclusions presented therein; (2) each author believes that the paper represents honest work; and (3) all authors have contributed to the present paper with equal effort.

## Conflict of Interest Statement

The authors declare that the research was conducted in the absence of any commercial or financial relationships that could be construed as a potential conflict of interest.
